# Evaluation of person-centered interventions to eliminate perinatal HIV transmission in Kisumu County, Kenya: A repeated cross-sectional study using aggregated registry data

**DOI:** 10.1371/journal.pmed.1004441

**Published:** 2024-08-15

**Authors:** Francesca Odhiambo, Raphael Onyango, Edwin Mulwa, Maurice Aluda, Linda Otieno, Elizabeth A. Bukusi, Craig R. Cohen, Pamela M. Murnane

**Affiliations:** 1 Centre for Microbiology Research, Research Care and Training Program, Kenya Medical Research Institute, Nairobi, Kenya; 2 Department of Obstetrics, Gynecology and Reproductive Sciences, University of California San Francisco, San Francisco, California, United States of America; 3 Bixby Center for Global Reproductive Health, University of California San Francisco, San Francisco, California, United States of America; 4 Institute for Global Health Sciences, University of California San Francisco, San Francisco, California, United States of America; 5 Department of Epidemiology and Biostatistics, University of California San Francisco, San Francisco, California, United States of America; University of Southampton, UNITED KINGDOM OF GREAT BRITAIN AND NORTHERN IRELAND

## Abstract

**Background:**

Following a decline in perinatal HIV transmission from 20% to 10% between 2010 and 2017 in Kenya, rates have since plateaued with an estimated 8% transmission rate in 2021. Between October 2016 and September 2021, Family AIDS Care & Education Services (FACES) supported HIV care and treatment services across 61 facilities in Kisumu County, Kenya with an emphasis on service strengthening for pregnant and postpartum women living with HIV to reduce perinatal HIV transmission. This included rigorous implementation of national HIV guidelines and implementation of 3 locally adapted evidence-based interventions targeted to the unique needs of women and their infants. We examined whether these person-centered program enhancements were associated with changes in perinatal HIV transmission at FACES-supported sites over time.

**Methods and findings:**

We conducted a repeated cross-sectional study of annually aggregated routinely collected documentation of perinatal HIV transmission risk through the end of breastfeeding at FACES-supported facilities between October 2016 and September 2021. Data included 12,599 women living with HIV with baseline antenatal care metrics, and, a separate data set of 11,879 mother–infant pairs who were followed from birth through the end of breastfeeding (overlapping with those in antenatal care 2 years prior). FACES implemented 3 interventions for pregnant and postpartum women living with HIV in 2019: (1) high-risk clinics; (2) case management; and (3) a mobile app to support treatment engagement. Our primary outcome was infant HIV acquisition by the end of breastfeeding (18 to 24 months). We compared infant HIV acquisition risk in the final year of the FACES program (2021) to the year before intervention scale-up and following implementation of the “Treat All” policy (2018). Mother–infant pair loss to follow-up was a secondary outcome. Program data were aggregated by year and site, thus in multivariable regression, we adjusted for site-level characteristics, including facility type, urban versus rural, number of women with HIV in antenatal care each year, and the proportion among them under 25 years of age. Between October 2016 and September 2021, 81,172 pregnant women received HIV testing at the initiation of antenatal care, among whom 12,599 (15.5%) were living with HIV, with little variation in HIV prevalence over time. The risk of infant HIV acquisition by 24 months of age declined from 4.9% (101/2,072) in 2018 to 2.2% (48/2,156) in 2021 (adjusted risk difference −2.6% [95% confidence interval (CI): −3.7, −1.6]; *p* < 0.001). Loss to follow-up declined from 9.9% (253/2,556) in 2018 to 2.5% (59/2,393) in 2021 (risk difference −7.5% [95% CI: −8.8, −6.2]; *p* < 0.001). During the same period, UNAIDS estimated rates of perinatal transmission in the broader Nyanza region and in Kenya as a whole did not decline. The main limitation of this study is that we lacked a comparable control group.

**Conclusions:**

These findings suggest that implementation of person-centered interventions was associated with significant declines in perinatal HIV transmission and loss to follow-up of pregnant and postpartum women.

## Abstract 2

Francesca Akoth Odhiambo and colleagues examined whether person-centered interventions were associated with changes in perinatal HIV transmission at FACES-supported sites in Kisumu County, Kenya.

## Author summary

**Why was this study done?:**

- Globally and in Kenya, ongoing perinatal HIV transmission persists at unacceptable rates despite the availability of highly effective treatment to prevent infant HIV acquisition.
- In the era of “Treat All,” limited programmatic exist that describe perinatal transmission through to the end of breastfeeding for mother–infant pairs in routine healthcare settings.
- The Family AIDS Care & Education Services (FACES) program aimed to eliminate perinatal HIV transmission via person-centered interventions to support vulnerable women in Kisumu Kenya.

**What did the researchers do and find?:**

- We used programmatic data from FACES to assess whether implementation of person-centered interventions for pregnant and postpartum women living with HIV was associated with reduced perinatal transmission and improved engagement in care between October 2016 through September 2021 in Kisumu County Kenya.
- We found that both 24-month perinatal transmission and loss to follow-up declined over the 5-year period when similar declines were not observed in the wider Nyanza region that encompasses Kisumu County.

**What do these findings mean?:**

- These findings suggest that person-centered services have the potential to meaningfully improve clinical outcomes for pregnant and postpartum women living with HIV and to reduce perinatal HIV transmission.
- The use of routine healthcare data enhances the generalizability of our findings to other high maternal HIV prevalence settings.
- The main limitation of this study is the lack of a similar control group that did not receive these interventions. Thus, the trends we observed could be due to factors not associated with the interventions.

## Introduction

Elimination of perinatal HIV transmission remains a global health priority [1]. Because antiretroviral treatment (ART) with adequate adherence during pregnancy and breastfeeding virtually eliminates transmission risk [2], the World Health Organization recommended lifelong ART for all pregnant and breastfeeding women living with HIV in 2013 (Option B/B+ [3]) and, subsequently, treatment of all persons regardless of age, sex, or CD4 count in 2016 (Treat All [4]). Global adoption of these recommendations dramatically increased the number of pregnant and breastfeeding women living with HIV accessing treatment and led to a significant decline in perinatal transmission [5]. However, declines have flattened in recent years, and advances have not been universal, with rates ranging between 2% in Botswana to 28% in the Democratic Republic of the Congo [5].

Globally, maternal disengagement from care is a leading driver of ongoing perinatal transmission [5]. Indeed, interventions to eliminate perinatal transmission are dependent upon the retention of women and their infants to ensure each step of the prevention of mother-to-child transmission (PMTCT) cascade is met, including maternal HIV testing, maternal ART for those living with HIV, infant antiretroviral prophylaxis, and infant HIV testing [6]. Based on prior literature, UNAIDs models of country-level perinatal transmission assume postpartum disengagement from HIV care averages 1.2% per month in the first 12 months and declines thereafter to 0.7% per month to the end of breastfeeding [7]. This may be conservative given that studies from low- and middle-income countries have reported approximately 40% attrition by 18 months of age with the majority occurring by 6 months postpartum [8]. Reported risk factors for disengagement include a new diagnosis of HIV, young maternal age, HIV-related stigma, unstable social support, and a history of unsuppressed viral load [9–12].

Kenya has made significant strides in antenatal service coverage with 97% of women accessing at least 1 visit, providing a window of opportunity for HIV testing and linkage to care [13]. Among women who became pregnant in 2017, 96% received HIV testing and among those seropositive, 92% received ART [13]. By 2017, the perinatal transmission rate was 10%, down from 20% in 2010, but only modestly declined to 8% by 2021 [5]. Over a third of ongoing perinatal transmission in Kenya is attributed to maternal disengagement from ART during pregnancy or breastfeeding [5].

Between 2016 and 2021, through Family AIDS Care & Education Services (FACES), we adapted and implemented 3 person-centered interventions to specifically target pregnant and breastfeeding women at risk of transmission or care disengagement. The interventions included establishing a high-risk clinic with individualized services, case management to tailor support and ensure rapid identification of new risk factors, and a mobile phone retention app. In this analysis, we sought to examine whether these person-centered program enhancements were associated with changes in perinatal HIV transmission at FACES-supported sites.

## Methods

### Ethics statement

The study was reviewed and approved by ethical review boards at the Kenya Medical Research Institute (#1/2009) and the University of California San Francisco (#11–05348). This activity was also reviewed by the United States Centers for Disease control and Prevention (CDC) and was conducted consistent with applicable federal law and CDC policy. Due to the nature of the retrospective and aggregated routine data, informed consent of persons included in this analysis was neither feasible nor required, as approved by the 3 institutional review boards. This study is reported as per the Strengthening the Reporting of Observational Studies in Epidemiology (STROBE) guideline (S1 STROBE Checklist).

### Overview

We conducted a repeated cross-sectional study of annually aggregated routinely collected data to quantify the risk of perinatal HIV transmission through the end of breastfeeding at FACES-supported facilities in Kisumu Country, Kenya, between October 2016 and September 2021. We hypothesized that perinatal transmission risk would be lower in 2021, the final program year, compared to 2018, the year prior to the implementation of person-centered care services.

### Study setting and population

This study includes all pregnant and postpartum women living with HIV who were followed at one of 61 FACES-supported Ministry of Health facilities in Kisumu Kenya between October 2016 and September 2021. FACES is a University of California San Francisco and Kenya Medical Research Institute partnership founded in 2004. Between October 2016 through September 2021, FACES was funded by the President’s Emergency Plan for AIDS Relief (PEPFAR)/Center for Disease Control & Prevention to provide technical support in the provision of HIV treatment and prevention programs in Kisumu County, located in the Nyanza Region of western Kenya bordering Lake Victoria. Kisumu County’s population is just over 1 million [14] with one of the highest HIV burdens in Kenya, including 21% HIV prevalence among women of childbearing age (15 to 49 years) in 2017 [13]. HIV prevalence in Kenya overall among adults age 15 to 49 years was 5% in 2017 [5].

### The FACES program

FACES aimed to strengthen health systems and enhance HIV care, treatment, and prevention services through leadership, mentorship, training, and oversight. In 2016, the FACES team intensified efforts to achieve meaningful progress toward the elimination of perinatal HIV transmission. We established annual county-level leadership meetings and monthly facility-level meetings to monitor outcomes among pregnant and postpartum women living with HIV, review clinical services and processes, ensure adequate human resources, and provide training and mentorship of health care workers. When outcomes fell short of expectations, we reviewed programmatic data to identify gaps in services and characteristics of vulnerable women to inform strategies for improvement.

As per the 2016 treatment guidelines, FACES ensured that all pregnant women attending antenatal care were offered opt-out HIV testing followed by same-day ART initiation if positive. HIV seronegative women received repeat HIV testing in the third trimester (≥27 weeks gestation) or at delivery and in the postnatal period, coupled with the provision of pre-exposure prophylaxis for those at risk of HIV acquisition. All women living with HIV were offered treatment adherence support at every visit and viral load monitoring every 6 months until complete cessation of breastfeeding. Their infants received HIV DNA polymerase chain reaction (PCR) tests at 6 to 8 weeks, 6 months and 12 months of age, and a rapid antibody test at 18 months or 6 weeks post cessation of breastfeeding, whichever was later. Infant prophylaxis was prescribed from 0 to 12 weeks of age (6 weeks of zidovudine plus nevirapine and nevirapine thereafter) or until the infant was determined to have acquired HIV. Updated national guidelines in 2018 recommended continuation of infant nevirapine prophylaxis until 6 weeks after breastfeeding cessation and an additional maternal viral load assessment 3 months after ART initiation for those initiating in the perinatal period.

Through our monthly data reviews and supported by the literature, we identified key drivers of ongoing perinatal transmission and disengagement from care, leading to our adoption of 3 person-centered interventions (Table 1). Briefly, these included: (1) a high-risk clinic day for women considered at risk of perinatal transmission or poor care engagement; (2) case management for 1:1 psychosocial support and close clinical monitoring; and (3) a mobile app to facilitate adherence support and mother–infant pair tracking. These interventions were planned and developed in 2018, implemented throughout 2019, and fully brought to scale by the end of 2019.

**Table 1:** FACES intervention descriptions.

Intervention	Description
High risk clinic	We established targeted clinics days for women considered at risk of perinatal transmission or poor care engagement to enable multidisciplinary team review of cases and to provide social networking for adolescents. Women with any of the following characteristics were eligible: • Newly diagnosed with HIV in the current pregnancy or breastfeeding • Adolescents aged 10–19 years • Unstable support system (stigma within the household or family, recent migration from home, food insecurity) • Any viral load >200 copies/ml in the last 12 months • History of poor adherence prior to the current pregnancy • Any history of a treatment interruption greater than 30 days Package of services provided at “high-risk clinics” • Moms’ Clubs for adolescents for peer support and safe and acceptable spaces for treatment interventions • Multidisciplinary review and patient management at every visit • Psychosocial and disclosure status reviewed at every visit by mentor mothers • Nutritional support
Case management	Case managers were charged with planning, obtaining, coordinating, and monitoring treatment needs of the individuals assigned to them. Case managers had at least a diploma in social science. Each followed roughly 10 individuals at any given time. The case manager provided the following support until the end of breastfeeding: • Developed treatment goals with individuals assigned to them based on their identified barriers • Developed an individual treatment plan which was reviewed regularly by the clinic team • Provided one-on-one psychosocial support where needed • Appointment tracking with phone reminders as needed
Mobile App	*Ushauri* is a mobile app for clinic use to support adherence, retention, and mother–infant pair tracking. The app was developed by mHealth Kenya and customized using feedback from user acceptance testing by FACES. The app included the following modules: • Digital appointment diary with automated same day alerts to case managers of any missed appointments • Automated appointment reminder messages sent 1 week before, 1 day before, and the morning of the appointment • Two-way text messaging with motivational messages to specific groups of individuals in care • Linked to medication pickup and clinical review to ensure completion of services

### Data sources

Data for this analysis primarily originated in paper-based registers that were domiciled at each of the supported health facilities. These data were aggregated at each health facility (quarterly for maternal and infant HIV testing and maternal ART uptake, and annually for 18 to 24 months infant outcomes) and compiled for PEPFAR annual reporting. The HIV-exposed infant cohort register tracked mother–infant pairs longitudinally from birth through the end of breastfeeding up to 24 months of age and included all infant HIV test results, mortality, and loss from care. Infant mortality was ascertained through chart abstraction or caregivers’ self-reports. Women who transferred in, initiated, or re-initiated HIV care were entered in the registers in their infants’ respective birth years. Infants who acquired HIV were exited from the HIV-exposed cohort and followed via HIV care and treatment registers. Therefore, deaths after HIV acquisition were not captured in these data. Mother–infant pairs >30 days late for a scheduled appointment visit were classified as lost to follow-up. These HIV-exposed infant cohort registry data were used for infant outcomes by 18 to 24 months each year (HIV acquisition, death, and loss to follow-up, detailed below), reflecting the outcomes of infants born 2 years prior. The antenatal care register tracked all maternal HIV tests and test results. The early infant diagnosis register captured all infant HIV-1 PCR tests before 12 months of age. In addition to register-based aggregated data, this analysis leverages individual-level viral load test results which are available for secure download from the National AIDS and Sexually Transmitted Infection Control Programme database. For women with more than 1 test result while pregnant or breastfeeding within a given year, the last result was retained.

We did not have access to a comparable control group that did not introduce FACES interventions. To assess the potential that any changes we observed at FACES were due to external trends other than the program-related interventions, we provided perinatal transmission rates from UNAIDS for Kenya overall and the Nyanza Region between 2017 and 2021, retrieved from AIDSinfo [5]. Notably, the populations for these estimates differ from our data as they incorporate both empirically reported data and predicted infant outcomes for women who either never engaged or disengaged from care. Therefore, we provided these numbers as a reference point and did not make statistical comparisons.

### Study outcomes

The primary study outcome was infant HIV acquisition by 18 to 24 months. For women still breastfeeding at 24 months (an estimated 1.5% of the population in this setting), final infant testing was delayed; these infants are excluded from this aggregated data. Secondary outcomes included mother–infant pair loss to follow-up, infant mortality, early infant HIV acquisition (<2 months), and maternal viremia during pregnancy and breastfeeding (plasma HIV RNA ≥1,000 copies/ml). Our facility-level aggregated data combined loss to follow-up with formal transfers out. However, the annual narratives that accompanied the data disaggregated the 2 but only at the overall program level.

### Primary exposure

While theoretically the exposure was the enhanced FACES services, our primary exposure variable was calendar year, with our primary comparison being a pre (2018)–post (2021) analysis.

### Covariates

We considered facility-level characteristics in adjusted models. These included facility type (dispensary, health center, hospital) and urban versus rural as time-fixed covariates. As time-varying covariates, each year we calculated the average number of women living with HIV in antenatal care, and among them the proportion under 25 years of age. Larger numbers of patients might reflect more experienced facilities with more services, or could reflect over-burdened facilities, either of which might influence outcomes. We note that the women in antenatal care were not the same women as those being assessed for perinatal transmission at 24 months postpartum in a given year; however, the population in antenatal care may reflect the general nature of services provided at that time. The viral load data set was individual level. For viral load analyses, we adjusted for maternal age (measured continuously) and treatment regimen, classified as efavirenz-containing, dolutegravir containing, or other, as well as facility type and urban versus rural.

### Statistical analysis

We reported the frequency of outcomes annually and estimated marginal differences in the outcomes by year via logistic regression models with robust variance estimation accounting for clustering by site. The primary exposure was a categorical variable for year with 2018 as the reference category. We tested our primary hypothesis that infant HIV acquisition in 2021 differed from 2018, with the coefficient for the year 2021 in the adjusted model. To model mortality and HIV infection by 24 months, we excluded those lost or transferred from the denominator to avoid underestimation of these outcomes and used multinomial logistic regression to estimate both outcomes in the same model. Because we lacked an external comparable control group and individual-level data for our primary outcome, we assessed whether facility-level characteristics, some of which changed over time, could have explained any changes in outcomes. Covariates are detailed above. All continuous variables were entered in models as linear terms.

### Analysis plan revisions

This study did not have a prospective analysis protocol. We made 3 changes to analytic plans during the conduct of this work. First, when we realized the potential impact of Treat All on outcomes between 2017 and 2018, we chose to use 2018 as the reference year for our analyses rather than 2017. In 2017, women starting antenatal care (ANC) living with HIV may not have had the opportunity to initiate ART prior to pregnancy due to higher CD4 counts. By 2018, Treat All was widely implemented and most women with known HIV status had initiated ART before antenatal care. Because our interventions were brought to scale throughout 2019, we deemed 2018 an appropriate “pre” year for a pre-post analysis. Second, in response to reviewer input, we now adjusted for facility-level characteristics in regression models and accounted for clustering by facility. Third, in a refreshed data download for the viral load analysis, we identified maternal age and treatment regimen in the data set and now included these variables in adjusted models.

## Results

**Fig 1:**
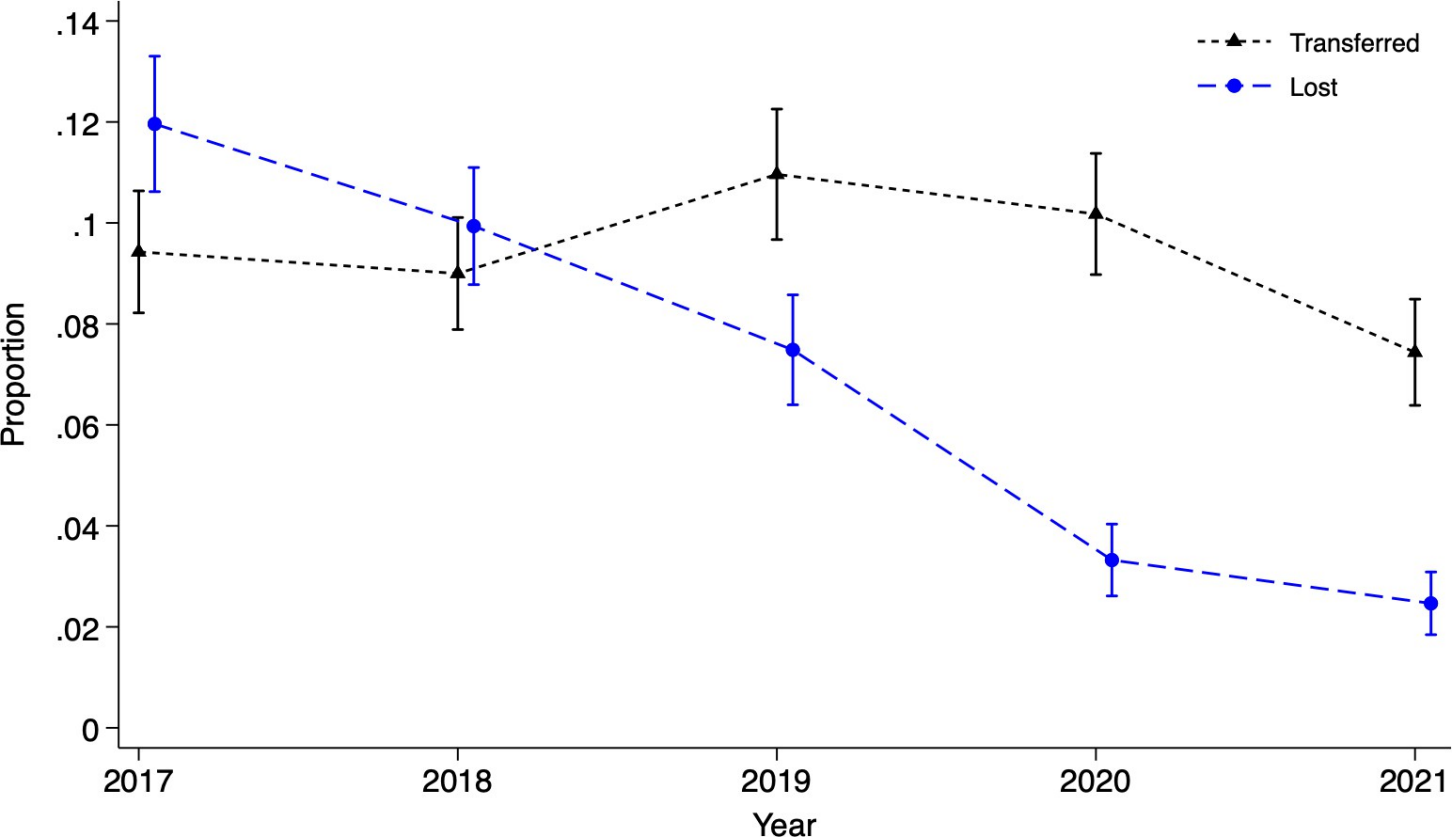
Proportion of mother–infant pairs lost to follow-up or transferred out with 95% CIs. The blue line represents the proportion lost (12.0%, 9.9%, 7.5%, 3.3%, and 2.5% by year) and the gray line represents the proportion formally transferred out (9.4%, 9.0%, 11.0%, 10.1%, and 7.4% by year).

Between October 2016 and September 2021, 81,172 women attended antenatal care and received HIV testing at 61 FACES-supported clinics. Most clinics were in rural settings (*n* = 56; 92%) with relatively small patient populations, though the average number of women living with HIV initiating antenatal care each month varied widely by facility (median 2; interquartile range 1, 3; range 0, 29). HIV prevalence was 15.5% overall with little variation over time (Table 2). Among women living with HIV initiating antenatal care, the proportion over age 25 years increased from 61.5% to 75.3% and the proportion newly diagnosed modestly declined from 23.6% to 17.4% between 2017 and 2021. Overall, ART access during pregnancy was 99.1%. Women and their infants who reached 18 to 24 months of age each year, an overlapping but not identical group to those in antenatal care 2 years prior, included 11,879 women living with HIV. Among them, loss to follow-up declined from 9.9% to 2.5% between 2018 and 2021 while there was little change in transfers out, an outcome we would not expect to change in response to our interventions (Fig 1). While loss to follow-up and transfers out were disaggregated in annual report narratives, they were combined in our facility-level data, which we used in regression analyses to account for facility level differences. Between 2018 and 2021, the adjusted risk difference of combined losses and transfers, largely driven by a reduction in loss to follow-up, was −8.5% (95% CI: −11.4, −5.7; *p* < 0.001).

**Table 2:** Characteristics of women attending antenatal care at FACES facilities during the study period, by year.

	Fiscal Year					
	2017	2018	2019	2020	2021	Total
***N* women in ANC with HIV testing**	14,256	17,875	16,555	16,902	15,584	81,172
HIV status						
Negative	11,939 (83.7%)	14,981 (83.8%)	14,022 (84.7%)	14,255 (84.3%)	13,376 (85.8%)	68,573 (84.5%)
Positive	2,317 (16.3%)	2,894 (16.2%)	2,533 (15.3%)	2,647 (15.7%)	2,208 (14.2%)	12,599 (15.5%)
**Among women with HIV in ANC**	*N* = 2,317	*N* = 2,894	*N* = 2,533	*N* = 2,647	*N* = 2,208	*N* = 12,599
Age in years*						
<20	160 (6.9%)	161 (5.6%)	139 (5.5%)	122 (4.6%)	119 (5.4%)	701 (5.6%)
20–24	732 (31.6%)	753 (26.0%)	586 (23.1%)	563 (21.3%)	426 (19.3%)	3,060 (24.3%)
25+	1,425 (61.5%)	1,978 (68.4%)	1,808 (71.4%)	1,962 (74.1%)	1,663 (75.3%)	8,836 (70.1%)
HIV status known at ANC entry						
Newly identified	546 (23.6%)	594 (20.5%)	532 (21.0%)	476 (18.0%)	385 (17.4%)	2,533 (20.1%)
Known	1,771 (76.4%)	2,300 (79.5%)	2,001 (79.0%)	2,171 (82.0%)	1,826 (82.6%)	10,069 (79.9%)
Accessed ART in ANC						
No	37 (1.6%)	18 (0.6%)	21 (0.8%)	20 (0.8%)	12 (0.5%)	108 (0.9%)
Yes	2,280 (98.4%)	2,876 (99.4%)	2,512 (99.2%)	2,627 (99.2%)	2,196 (99.5%)	12,491 (99.1%)
**Among women with VL in pregnancy**	*N* = 217	*N* = 998	*N* = 3,248	*N* = 1,811	*N* = 825	*N* = 7,099
ART regimen core medication						
EFV based	118 (54.4%)	679 (68.0%)	2,078 (64.0%)	1,254 (69.2%)	103 (12.5%)	4,232 (59.6%)
DTG based	0 (0.0%)	2 (0.2%)	329 (10.1%)	369 (20.4%)	653 (79.2%)	1,353 (19.1%)
Other	99 (45.6%)	317 (31.8%)	841 (25.9%)	188 (10.4%)	69 (8.4%)	1,514 (21.3%)

* *n* = 2 are missing age in 2018.

ANC, antenatal care; ART, antiretroviral treatment; DTG, dolutegravir; EFV, efavirenz; FACES, Family AIDS Care & Education Services; *N*, number; VL, viral load.

### Infant outcomes

**Fig 2:**
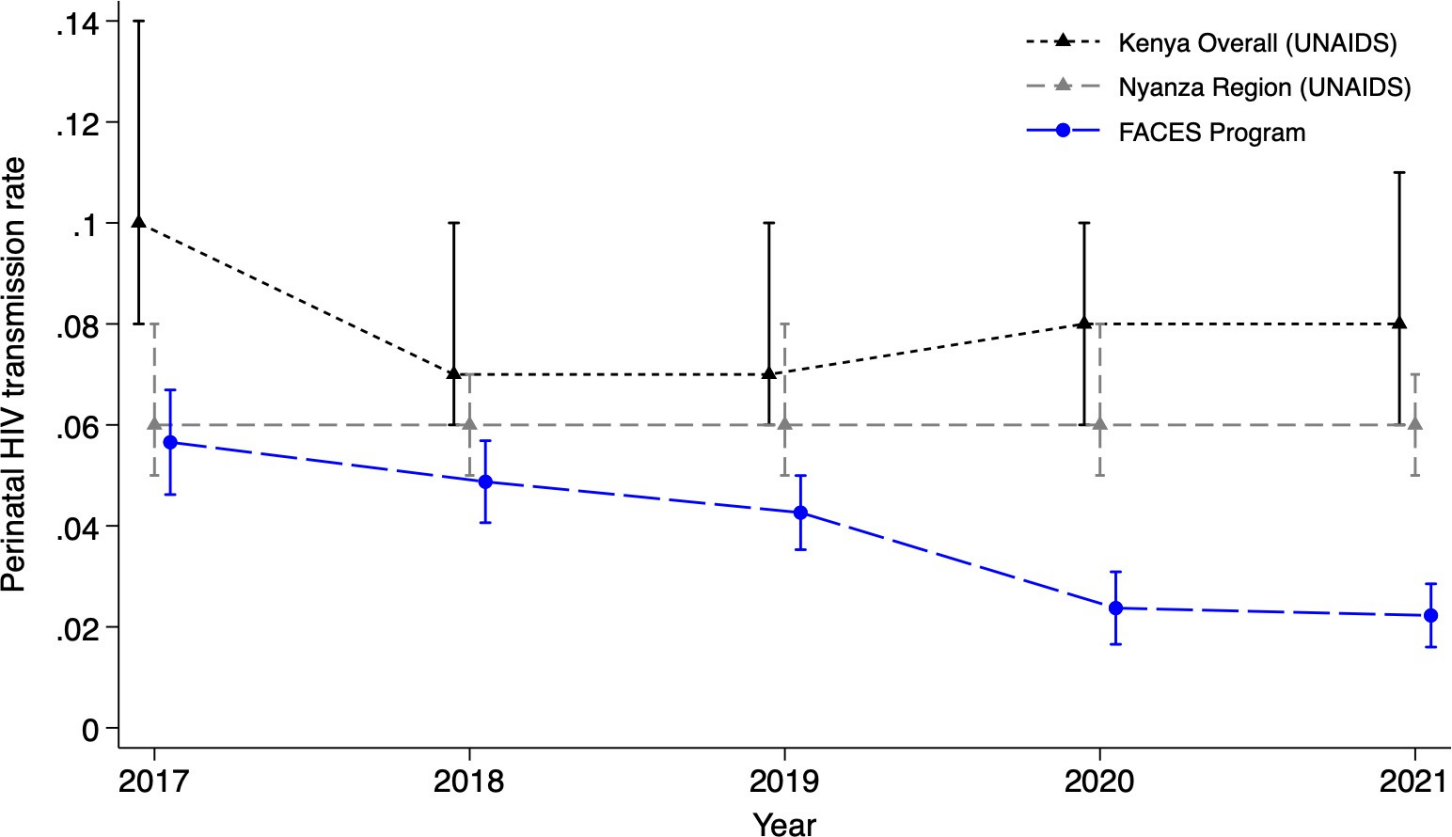
Perinatal HIV transmission from pregnancy through the end of breastfeeding at FACES sites (from programmatic data) and overall in Kenya and the Nyanza region (from UNAIDS) with 95% CIs. The blue line represents the FACES program, the solid gray line represents Kenya overall, and the dashed gray line represents the Nyanza region in Kenya.

Our primary study outcome, the cumulative risk of perinatal HIV transmission through the end of breastfeeding (18 to 24 months), declined from 4.9% in 2018, before FACES interventions were implemented, to 2.2% in 2021 (adjusted risk difference [RD] −2.6% [95% confidence interval, CI: −3.5, −1.8], *p* < 0.001) (Table 3). During the same period, a similar decline was not observed in the Nyanza region nor in Kenya overall (Fig 2). We also observed a 0.9% decline in in utero and early postpartum transmissions (<2 months) among infants receiving this early test (95% CI: −1.5, −0.3; *p* = 0.002). Among HIV-exposed and uninfected infants, the risk of mortality by 18 to 24 months declined from 2.8% in 2018 to 1.9% in 2021, though after adjusting for facility characteristics the difference was not statistically significant (adjusted RD −0.8% [95% CI: −1.7, 0.1]; *p* = 0.10).

**Table 3:** HIV-exposed infant outcomes at FACES-supported facilities between 2017 and 2021.

					Absolute risk differences				
	Year	*N*	*n* (%)	Unadjusted		*p*-Value	Adjusted	*p*-Value
**Infant HIV testing before 2 months of age**									
Early infant HIV acquisition	2017	2,003	57 (2.8%)	1.3% (0.6, 2.0)		0	1.2% (0.3, 2.1)	0.01
	2018	2,030	31 (1.5%)	*(reference group)*				
	2019	2,000	18 (0.9%)	−0.6% (−1.2, −0.1)		0.03	−0.6% (−1.2, −0.1)	0.03
	2020	2,094	22 (1.1%)	−0.5% (−1.1, 0.1)		0.12	−0.5% (−1.1, 0.2)	0.17
	2021	1,274	8 (0.6%)	−0.9% (−1.5, −0.3)		0.002	−0.9% (−1.5, −0.3)	0.002
**Final infant outcomes at the end of breastfeeding**									
**HIV**	2017	1,768	100 (5.7%)	0.8% (−0.4, 2.0)		0.21	0.7% (−0.8, 2.1)	0.36
	2018*	**2,072**	**101 (4.9%)**	*(reference group)*				
	2019	1,830	78 (4.3%)	−0.6% (−1.8, 0.5)		0.30	−0.6% (−1.9, 0.7)	0.35
	2020	2,108	50 (2.4%)	−2.5% (−3.7, −1.3)		<0.001	−2.5% (−3.8, −1.2)	<0.001
	2021*	**2,156**	**48 (2.2%)**	−**2.6% (**−**3.5,** −**1.8)**		**<0.001**	−**2.6% (**−**3.7,** −**1.6)**	**<0.001**
Death	2017	1,768	44 (2.5%)	−0.3% (−1.5, 0.9)		0.61	−0.4% (−1.5, 0.6)	0.437
	2018	2,072	58 (2.8%)	*(reference group)*				
	2019	1,830	53 (2.9%)	0.1% (−0.9, 1.1)		0.85	0.2% (−0.8, 1.2)	0.703
	2020	2,108	34 (1.6%)	−1.2% (−2.0, −0.4)		0.002	−1.0% (−1.9, −0.2)	0.02
	2021	2,156	40 (1.9%)	−0.9% (−1.7, −0.1)		0.02	−0.8% (−1.7, 0.1)	0.10
Lost or transferred	2017	2,249	481 (21.4%)	2.5% (−0.6, 5.5)		0.12	2.1% (−1.2, 5.3)	0.21
	2018	2,556	484 (18.9%)	*(reference group)*				
	2019	2,244	414 (18.4%)	−0.5% (−3.4, 2.4)		0.74	−0.2% (−3.2, 2.8)	0.90
	2020	2,437	329 (13.5%)	−5.4% (−7.7, −3.1)		<0.001	−5.0% (−7.7, −2.3)	<0.001
	2021	2,393	237 (9.9%)	−9.0% (−11.4, −6.7)		<0.001	−8.5% (−11.4, −5.7)	<0.001

* HIV acquisition by the end of breastfeeding is the primary study outcome; the contrast between 2021 and 2018 examines the hypothesis that rates declined over that period.

Early infant HIV tests (<2 months of age) are from the early infant testing register and reflect tests done within the fiscal year; final outcomes are from the longitudinal HIV-exposed infant register and reflect infants who reached 18–24 months within the fiscal year. HIV and mortality denominators exclude those lost/transferred to avoid underestimation.

*N*, total number of HIV-exposed infants included in the analysis each year; *n*, the number of HIV-exposed infants with the outcome each year; FACES, Family AIDS Care & Education Services.

### Maternal viral load

The number of women receiving virologic monitoring during pregnancy and breastfeeding significantly increased in 2019 following implementation of the 2018 guidelines which added an additional test in pregnancy for those newly diagnosed and also resulted in overall reinforcement of viral load monitoring according to schedule (Table 4). Because most women breastfeed for nearly 2 years, nearly twice the number of pregnant women would be eligible for a viral load test during breastfeeding each year. In 2021, supply chains were significantly disrupted, and viral load monitoring nearly came to a halt for much of the year. Among women who received viral load testing during pregnancy, 6.4% and 2.9% had viremia (HIV RNA ≥1,000 copies/ml) though this difference was no longer significant after adjusting for facility characteristics, maternal age, and treatment regimen (adjusted RD: −1.3% [95% CI: −3.3, 0.6]; *p* = 0.18). Results during breastfeeding were comparable.

**Table 4:** Maternal viremia (plasma HIV RNA ≥1,000 copies/ml) at FACES-supported facilities between 2017 and 2021.

			Absolute risk differences			
Pregnant	*N*	*n* (%)	Unadjusted	*p*-Value	Adjusted	*p*-Value
2017	217	20 (9.2%)	2.8% (−2.0, 7.6)	0.25	2.1% (−1.5, 5.7)	0.26
2018	998	64 (6.4%)	*(reference)*			
2019	3,248	171 (5.3%)	−1.1% (−2.5, 0.2)	0.10	0.1% (−1.0, 1.2)	0.83
2020	1,811	94 (5.2%)	−1.2% (−2.9, 0.5)	0.16	0.0% (−1.4, 1.5)	0.98
2021	825	24 (2.9%)	−3.5% (−5.1, −1.9)	<0.001	−1.3% (−3.3, 0.6)	0.18
Breastfeeding						
2017	447	46 (10.3%)	3.0% (−0.5, 6.6)	0.10	2.2% (−0.6, 5.1)	0.12
2018	1,909	139 (7.3%)	*(reference)*			
2019	3,446	187 (5.4%)	−1.9% (−3.5, −0.2)	0.03	−0.9% (−2.3, 0.5)	0.23
2020	4,063	173 (4.3%)	−3.0% (−4.7, −1.3)	<0.001	−1.2% (−2.8, 0.3)	0.12
2021	2,277	66 (2.9%)	−4.4% (−6.0, −2.7)	<0.001	−1.2% (−3.3, 0.9)	0.26

Data include the last test of the fiscal year per person if >1 test was done.

*N* total number of with a viral load test included in the analysis each year; *n*, the number of women with viremia each year; FACES, Family AIDS Care & Education Services.

## Discussion

In this repeated cross-sectional study from a large HIV care and treatment program in Kisumu County, the risk of perinatal transmission by the end of breastfeeding declined by more than half between 2018 and 2021 to 2.2%, while postpartum loss to follow-up and viral non-suppression declined by more than half to 2.5% and 2.9%, respectively. These declines were associated with optimized implementation of national guidelines to prevent perinatal transmission and scale-up of person-centered interventions. Dolutegravir-containing ART, which has been associated with less virological failure or drug resistance and higher infant survival [15] was also scaled up Kenya-wide during this period. Our observed decline in viral non-suppression over time was attenuated after adjusting for regimen, though we did not have individual-level data to examine regimen in association with perinatal transmission. Notably, during the same time period, Kenya-wide and Nyanza region estimates of perinatal transmission did not meaningfully change, suggesting that the sharp declines in perinatal transmission that we observed at FACES-supported sites was not fully explained by dolutegravir scale-up. Furthermore, the Nyanza region is a high priority area for HIV control which is reflected in the lower than national perinatal transmission rates, yet the FACES estimates still diverge from the Nyanza over the 5-year period. Finally, the concurrent declines in loss to follow-up suggest that the enhanced FACES services resulted in improved patient engagement which is the cornerstone of prevention of perinatal transmission.

The perinatal HIV transmission rate at 18 to 24 months postpartum in our study is among the lowest reported from recent cohort studies in predominantly breastfeeding populations, consistent with findings of 2.9% among 613 mother–infant pairs in Lesotho [16] and 2.2% among 608 mother–infant pairs in Rwanda [17]. However, observational cohorts tend to offer closer follow-up than routine care and require a willingness to consent and participate. As such, infants at high risk of HIV acquisition may be underrepresented and perinatal transmission in routine care in these settings could be higher. In Tanzania, an analysis of individual-level routine care data found 1.4% transmission among 13,251 mother–infant pairs, though authors noted significant missing data, as in many other studies of individual-level routine care [18]. Each of these studies reported near universal ART coverage, comparable to our findings. In contrast, studies from Malawi [19] and South Africa [20] that observed perinatal transmission by 18 to 24 months in 4.9% and 4.3%, respectively, also reported less than 85% ART coverage.

Prior evidence has indicated that key drivers to loss to follow-up and perinatal transmission include young age, incident infection, and a history of viral non-suppression [9–12]. At FACES, women <25 years of age, those newly diagnosed with HIV in the current pregnancy, and those with elevated viral loads comprised 30%, 20%, and 5% of the study population, respectively. We specifically targeted these populations with a package of evidence-based retention strategies [21–23] and observed significant improvements in retention in care concurrent with these programs. A recent systematic review found that stand-alone interventions had only modest effects on perinatal HIV transmission, while integrated packages of services had higher effectiveness, addressing a variety of challenges that women face [24]. We, too, introduced an integrated package of services to enhance the potential to reach the most vulnerable.

Mortality among HIV-exposed infants modestly declined from 2.8% to 1.6%, with notably low mortality at the start of the study period, lower than the 4% to 6% found in the Lesotho and Rwanda studies mentioned above. However, our estimates are based on mother–infant pairs tracked in the HIV-exposed infant register and therefore may exclude infants who died within the first days of life.

Major strengths of our study include the use of a large programmatic cohort from both rural and urban settings and high-quality data from a real-world clinical setting, enhancing the generalizability of our findings to routine ART delivery among similar populations with high HIV prevalence. The main limitation of our study is the lack of a comparable control group of women who engaged with clinical care and did not receive the FACES interventions during the same time period. Additionally, we were not able to assess perinatal transmission among mother–infant pairs who were lost to follow-up or who transferred their care out of FACES-supported health clinics. Mother–infant pairs that are lost to follow-up have an increased risk of transmission, though notably by 2021 only 2.5% were classified as lost. Furthermore, we lacked individual-level data for the analysis of perinatal transmission and could not directly assess whether changes in the study population, clinical or otherwise, differed across the mother–infant pair cohorts. Finally, because our program enhancements and interventions were planned and delivered in response to clinical needs and were not originally designed as research activities, we did not collect data on fidelity or other implementation outcomes. Through our annual county-level monthly facility-level meetings, we carefully reviewed clinical services and outcome data to optimize fidelity. We expect this process could be further streamlined and provide greater insight into gaps in care as more facilities adopt electronic medical record systems.

In conclusion, following the introduction of person-centered, locally driven solutions for women at high risk of care disengagement coupled with rigorous implementation of national policies, FACES-supported health facilities nearly reached elimination of perinatal HIV transmission by 2021, demonstrating the possibility of reaching UNAIDS elimination targets (<2%) in predominantly breastfeeding populations. In 2020, UNICEF published a roadmap for the elimination of perinatal HIV transmission, recommending many similar strategies and approaches used by FACES [25]. Based on our experience, we believe that locally driven strategies to optimize implementation of Kenya’s national guidelines, coupled with person-centered services for high-risk groups, have the potential to reduce the unacceptable ongoing rates of perinatal transmission across the country. The development of a risk stratification toolkit, which has been successfully used to guide the integration of HIV care and family planning [26], may help to standardize implementation while ensuring replicability and impact in similar settings.

## Supporting information

S1 STROBE Checklist STROBE Statement—checklist of items that should be included in reports of observational studies. (DOCX)

## Abbreviations

ART: antiretroviral treatment
CI: confidence interval
FACES: Family AIDS Care and Education Services
PCR: polymerase chain reaction
PMTCT: prevention of mother-to-child transmission
RD: risk difference
